# Desert dust episodes during pregnancy are associated with increased preterm delivery in French Guiana

**DOI:** 10.3389/fpubh.2024.1252040

**Published:** 2024-02-28

**Authors:** Mathieu Nacher, Malika Leneuve, Celia Basurko, Alphonse Louis, Dominique Dotou, Stephanie Bernard, Kathy Pannechou, Karim Merad Boudia, Lindsay Osei, Fabrice Quet, Najeh Hcini

**Affiliations:** ^1^CIC INSERM 1424, Centre Hospitalier de Cayenne, Cayenne, French Guiana; ^2^Amazonian Infrastructures for Population Health, Cayenne, French Guiana; ^3^Département Formation Recherche Santé, Université de Guyane, Cayenne, French Guiana; ^4^Service de Gynecologie Obstétrique, Centre Hospitalier de Cayenne, Cayenne, French Guiana; ^5^Réseau Périnatalité, Cayenne, French Guiana; ^6^ATMO-Guyane, Cayenne, French Guiana; ^7^Protection Maternelle et Infantile, Cayenne, French Guiana; ^8^Service de Gynécologie obstétrique, Centre Hospitalier de l’Ouest Guyanais, Saint Laurent du Maroni, French Guiana

**Keywords:** preterm birth, air pollution, airborne particulate matter, seasons, French Guiana

## Abstract

Preterm deliveries are a major multifactorial public health problem in French Guiana. Desert dust episodes have been associated with preterm delivery in Guadeloupe, a territory with similarities to French Guiana. We thus tried to replicate this finding in the context of French Guiana. A retrospective ecological cohort study combined daily PM10 concentration measurements during pregnancy and term at delivery extracted from French Guiana’s computerized pregnancy delivery registry. Daily PM10 concentrations during the course of pregnancy were analyzed as mean concentrations and as the proportion of intense dust episodes (≥55 μg PM_10_/m^3^). These exposure variables were studied in relation to the outcome of preterm delivery. Overall, 3,321 pregnant women with complete daily PM10 measurements were included, of whom 374 (11.26%) delivered prematurely. Among preterm deliveries, 168 (44.9%) were spontaneous deliveries and 206 (55.1%) were induced. Rank-sum tests showed that, for spontaneous and induced spontaneous deliveries, both mean PM10 concentrations and proportions of intense desert dust episodes were significantly greater among preterm births than among term births. Although the proportion of intense desert dust episodes during pregnancy was significantly associated with spontaneous preterm deliveries, the relation was U-shaped, with an adjusted odds ratio (AOR) = 2 (95%CI = 1.2–3.1) for lowest values relative to median values and AOR = 5.4 (95%CI = 3.2–8.9) for the highest values relative to median values. Similarly, the proportion of intense desert dust episodes during pregnancy was also significantly associated with induced preterm deliveries in a U-shaped manner (AOR = 2.7 (95%CI = 1.6–4.5) for the lowest relative to median values and AOR = 6.8 (95%CI = 3.9–11.9) for the highest relative to median values). Although in our study the relation between PM10 concentrations appeared non-linear, the highest mean concentrations and intense desert dust episodes were indeed associated with both spontaneous and induced preterm delivery.

## Introduction

Desert dust leads to the presence of large quantities of atmospheric particulate matter (PM) in many regions of the world. Although there are many studies about the health impacts of industrial and urban atmospheric pollution and particulate matter, the study of the impact of desert dust episodes on human health has been inconsistent. In Europe and the Caribbean, although some studies have linked sandy dust storms to a range of adverse health outcomes, including respiratory and cardiovascular conditions and mortality, others have found no harmful effect (1–8). Others still found an association between desert dust and neonatal mortality (9).

Anthropogenic particulate pollution is known to be associated with preterm birth (10–12). In Guadeloupe, because of regular trade winds and minimal heavy industrialization, PM10 concentrations are mainly due to Sahara desert dust episodes. Although the health impact of desert dust has been debated (13), in Guadeloupe in 2019, Viel et al. hypothesized that PM10 from desert dust may be related to preterm births. Hence, they observed a statistically significant association between desert dust episodes and preterm delivery (10). In 2011, in Barcelona, Spain, although there was no relation with other pregnancy outcomes, Dadvand et al. (13) found a small but significant association between the number of desert dust episodes and the gestational age at delivery. In another study in 2020 in nine Spanish regions, Moreira et al. also found significant relations between desert dust episodes and preterm births (14).

Moreira et al. (14) hypothesized that the relation between desert dust episodes or PM10 pollution and preterm delivery was linked to oxidative stress, pro-inflammatory, and pro-thrombotic stress, which lead to gestational hypertension, placental hypoperfusion, and dysfunction. Vadillo-Ortega et al. (15) hypothesized that inflammation affects the choriodecidual microenvironment and may trigger signaling networks with initial inflammation signals activating secondary compounds such as prostaglandins, oxytocin, and matrix metalloprotease, which would lead to cervical ripening, uterine contractions, and rupture of the fetal membranes. The authors also hypothesized that additional factors, such as genetic background (16, 17), obesity, and inflammation, may modulate this process. Apart from statistical noise or differences in study design, this could hypothetically explain why studies in populations of different ancestries with different prevalences of other risk factors yield contrasted results (10, 13, 14).

Like Guadeloupe, French Guiana is a French overseas territory with a largely Afro-descendent population, high rates of poverty, and a high incidence of preterm deliveries. The incidence of preterm deliveries in French Guiana is nearly double that of mainland France (13% vs. 7%, respectively) (18). Poverty, poor follow-up, and preeclampsia are among the common factors that underlie this, but there are still a lot of unknowns behind this excess preterm delivery (19). French Guiana is situated between Brazil and Suriname. As in Guadeloupe, trade winds and minimal industrialization are also found in French Guiana, and thus, PM10 concentrations are also largely influenced by desert dust. Between December and May, people in French Guiana are periodically exposed to desert dust from the North-Eastern Sahara transported along the northern margin of the intertropical convergence zone, where northern and southern trade winds converge along the thermal equator (20). This phenomenon fluctuates, sometimes peaking for several consecutive days above the health-based standards for PM ≤ 10 μm (PM10). Given the recent finding of an association between desert dust episodes and preterm delivery in Guadeloupe (10), we tried to replicate this finding in the context of French Guiana. The aim of the present study was thus to study the relation between desert dust episodes throughout pregnancy and their potential relation with preterm delivery in French Guiana.

## Methods

### Study design

The study design was an observational retrospective ecological cohort.

### Pregnancy data

The data from the delivery registry in Cayenne Hospital {RIGI [Registre d’Issue de Grossesses Informatisé, described in ref. Leneuve-Dorilas et al. (18)]} were studied for women with dates of conception ranging from December 2018 to March 2021 living and delivering in Cayenne. Cayenne Hospital maternity is the only maternity in the Cayenne area, and it is also the only level-3 maternity in French Guiana. In order to avoid biases due to transfers of complicated pregnancies from other cities, we used the residence location variable (available in the RIGI) and restricted inclusions to women living in Cayenne. The included women thus represented an exhaustive description of deliveries in Cayenne by women from Cayenne. All live-birth deliveries are systematically included in the registry; there is no selection.

Daily PM 10 data over Cayenne was obtained from ATMO-Guyane, the structure that monitors air quality in French Guiana (21). The method used to measure daily PM10 levels rests on microbalance radiometry [Tampered Element Oscillating Microelement-Filter Dynamics Measurement System (22)] collected from the CAIENAIII fixed station in Cayenne (Supplementary Figure S1). Measurements are reported daily before 2 p.m. ATMO-Guyane follows strict national guidelines and, as with all ATMO stations in France, complies with French law (the “code de l’environnement”). ATMO stations must be recertified every 3 years by the state, as required by decree n°2019-1341 issued 12 December 2019.

### Data analysis

Births were classified as premature or not. Premature births were defined as deliveries after 22 weeks and before 37 weeks of pregnancy. Miscarriages were not included because the RIGI registry only includes live births. We only analyzed observations for whom we had PM10 measurements throughout pregnancy, with at most 1 week of missing measurements (3,321 pregnancies). First, we compared the mean PM10 concentration throughout pregnancy and the proportion of intense dust episodes (≥55 μg PM_10_/m^3^) between preterm deliveries and term deliveries (including early-term, full-term, late-term, and post-term births). Our analysis was then aligned with that of the Guadeloupe study with two variables: (1) daily PM_10_ concentrations averaged over the entire pregnancy and (2) the proportion of days with intense dust episodes (≥55 μg PM_10_/m^3^) over the course of pregnancy. *Z*-scores of the two main exposure variables were categorized in order to align with the study in Guadeloupe reporting odds ratios for 1 SD increments. Crude odds ratios and adjusted odds ratios were computed using prematurity as the outcome variable and different predictors as independent variables. Multivariate analysis was performed to adjust for potential confounding by gravidity, parity, age, preeclampsia, trimester of the first consultation, and prenatal interview (the aim of the mandatory early prenatal interview, usually during the fourth month of pregnancy, is to enable the healthcare professional to assess the psychological, emotional, and social needs for better support during pregnancy). The variables selected in the model were those known to be associated with prematurity in the medical literature and those significantly linked in the bivariate analysis (for qualitative variables, cross-tabulations between the outcome and each variable or, for quantitative variables, rank sum tests). Because the mean PM10 concentration and the proportion of intense dust episodes were highly correlated (Spearman’s rho = 0.94), we did not put them in the same multivariate model to avoid multicollinearity. The statistical significance threshold was *p* < 0.05.

### Ethical and regulatory aspects

The pregnancy registry is a certified anonymized registry that has been operating for 25 years. It has been approved by the Regulatory Authority Commission Nationale Informatique et Libertés [CNIL (2,228,099 v 0)] and has led to numerous publications. The use of atmospheric and anonymized pregnancy outcome data did not require additional authorizations.

## Results

Overall, 3,321 pregnant women with complete PM 10 measurements were included, of whom 374 (11.26%) delivered prematurely. Among preterm deliveries, 168 (44.9%) were spontaneous deliveries the rest being induced.

**Figure 1:**
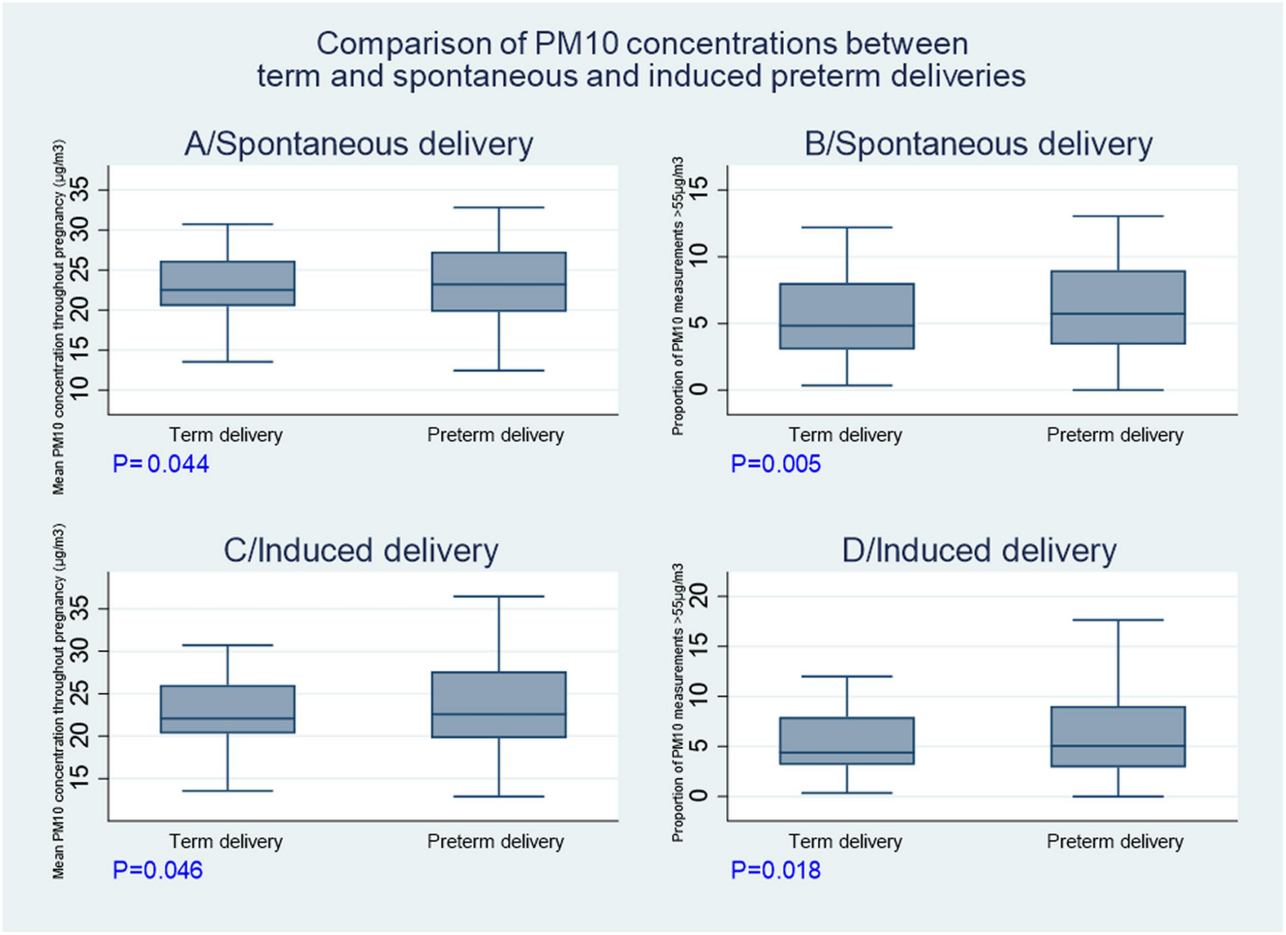
Comparisons of PM10 concentrations between term and preterm births.

Figures 1A–D shows the significant differences (rank sum tests) between induced and spontaneous term and preterm deliveries for PM10 mean concentrations throughout pregnancy and the proportion of intense dust episodes throughout pregnancy. For induced births, PM10 mean concentrations were greater in preterm deliveries than in term deliveries (23.1 ± 5.5 μg PM_10_/m^3^ vs. 22.5 ± 4.1 μg PM_10_/m^3^, respectively). For induced births, the proportion of intense desert dust episodes was greater in preterm deliveries than in term deliveries (6 ± 4.1% vs. 5.3 ± 2.9%, respectively). For spontaneous births, PM10 mean concentrations were greater in preterm deliveries than in term deliveries (23.1 ± 4.9 μg PM_10_/m^3^ vs. 22.7 ± 4.1 μg PM_10_/m^3^, respectively). For spontaneous births, the proportion of intense desert dust episodes was greater in preterm deliveries than in term deliveries (5.9 ± 3.5% vs. 5.4 ± 2.9%, respectively).

Table 1 shows the proportions of spontaneous and induced preterm deliveries by 1 SD category of PM10 mean concentrations throughout pregnancy and by 1 SD category of the proportion of intense dust episodes throughout pregnancy. Contrary to quantitative analysis, the table suggests a non-linear U-shaped relation. Table 1 also shows the proportions of spontaneous and induced deliveries for different categorizations of PM10 measurements. The most striking associations were observed between preterm delivery and the proportion of intense desert dust episodes. Table 2 shows that crude and adjusted odds ratios for the probability of spontaneous and induced preterm deliveries followed the same U-shaped relation for categorized z-scores of PM10 mean concentrations throughout pregnancy and for categorized z-scores of the proportion of intense dust episodes throughout pregnancy.

**Table 1:** Relation between categorized z-scores for PM10 measurements and spontaneous and induced preterm delivery.

	Preterm delivery *N* (%)	Term delivery* *N* (%)
*Spontaneous delivery*		
Categorized *z*-scores for proportion > 55 μg PM_10_/m^3^		
<0	76(7.1)	994 (92.9)
[0—1]	26(3.8)	661 (96.2)
>1 SD	55(17.9)	252 (82.1)
Categorized *z*-scores for mean PM_10_/m^3^		
<−1 SD	33 (9.7)	307 (90.3)
[−1 SD—0]	47 (6.5)	673 (93.5)
[0—1 SD]	40 (6.2)	606 (93.8)
>1 SD	48 (12.3)	341 (87.6)
*Induced delivery*		
Categorized *z*-scores for proportion > 55 μg PM_10_/m^3^		
<0	101 (15.7)	543 (84.3)
[0—1]	23 (6.7)	322 (93.3)
>1 SD	66 (31.4)	144 (68.6)
Categorized *z*-scores for mean PM_10_/m^3^		
<−1 SD	42 (19)	179 (81)
[−1 SD—0]	63 (14.9)	361 (85.1)
[0—1 SD]	38 (10.7)	318 (90.3)
>1 SD	63 (28)	162 (72)

* > =37 weeks pregnancy.

**Table 2:** Crude and adjusted odds ratios quantifying the relation between categorized *z*-scores for PM10 measurements and spontaneous and induced preterm delivery.

	Crude odds ratio (95% CI)	Adjusted* odds ratio (95% CI)	Wald *P*
*Spontaneous delivery*			
Categorized *z*-scores for proportion > 55 μg PM_10_/m^3^			
<0	1.9 (1.2–3.1)	2 (1.2–3.1)	*P* = 0.005
[0—1]	Ref	Ref	
>1 SD	5.5 (3.4–9.0)	5.4 (3.2–8.9)	*P* < 0.001
Categorized *z*-scores for mean PM_10_/m^3^			
<−1 SD	1.6 (1–2.6)	1.7 (1–2.7)	*P* = 0.04
[−1 SD—0]	1 (0.7–1.6)	1 (0.7–1.6)	*P* = 0.8
[0—1 SD]	Ref	Ref	
>1 SD	2.1 (1.4–3.3)	2 (1.3–3.2)	*P* = 0.002
*Induced delivery*			
Categorized *z*-scores for proportion > 55 μg PM_10_/m^3^			
<0	2.6 (1.6–4.2)	2.7 (1.6–4.5)	*P* < 0.001
[0—1]	Ref	Ref	
>1 SD	6.4 (3.8–10.7)	6.8 (3.9–11.9)	*P* < 0.001
Categorized *z*-scores for mean PM_10_/m^3^			
<−1 SD	1.9 (1.2–3.2)	2.1 (1.3–3.6)	*P* = 0.005
[−1 SD—0]	1.5 (0.9–2.2)	1.8 (1.1–2.9)	*P* = 0.01
[0—1 SD]	Ref	Ref	
>1 SD	3.2 (2.1–5.1)	3.7 (2.3–6.0)	*P* < 0.001

*Multiple logistic regression model with presence or absence of preterm delivery (<37 weeks) adjusting for gravidity, parity, age, preeclampsia, trimester of first consultation, and prenatal interview.

### Attributable fractions

We computed the population-attributable fractions for the highest category vs. others. The values were 16% for mean PM10 concentrations and induced preterm delivery; this means that 16% of the induced preterm deliveries in this population could have been eliminated if exposure to mean PM10 concentrations above 1 SD (>42.48 μg PM_10_/m^3^) were eliminated. For the proportion of intense desert dust episodes and induced preterm delivery, the population attributable fraction suggested that 21.6% of the induced preterm deliveries in this population could have been eliminated if this exposure had been eliminated. For spontaneous preterm delivery, the population attributable fractions were 12.8% for intense desert dust episodes, implying that 12.8% of the spontaneous preterm deliveries in this population could have been eliminated if this exposure had been eliminated. Finally, 5.7% of the spontaneous preterm deliveries in this population could have been eliminated if exposure to mean PM10 concentrations above 1SD (>42.48 μg PM_10_/m^3^) were eliminated.

### Breakdown by pregnancy trimester

When conducting sub-analyses by trimester of pregnancy, there were no significant differences in mean PM10 concentrations during the trimester between preterm and term deliveries (data not shown); however, the proportion of intense desert dust episodes during the third trimester was significantly greater in women with preterm delivery (9.2% for values below the mean, 5.9% for those between the mean and 1 SD, and 11.6% for those >1SD, *p* = 0.01).

## Discussion

Although there were significant differences between preterm and term deliveries regarding PM10 mean concentrations and severe dust episodes, we could not replicate any clear linear relation between dust episodes and increased risk for preterm delivery. However, in Guadeloupe, each 1 SD increase in PM10 was associated with a greater risk of preterm delivery; what was observed in French Guiana was instead a U-shaped relationship with the lowest PM10 concentrations, and PM10 concentrations greater than 1 SD were associated with a greater risk of both spontaneous and induced preterm delivery. The U-shape would suggest that too-low concentrations are bad, and too-high concentrations are also bad compared to an optimal level.

We have no clear explanation of why our U-shaped results are different from the more linear observations in Guadeloupe. Perhaps the high frequency of micronutrient deficiencies in French Guiana is such that some frequent deficiencies would be corrected by air particles (for example, airborne particles may contain Vitamin D and carotenoids, which may be absorbed through the lungs), but this is quite speculative (23–28). The computation of the population-attributable fractions of the greatest values for different measures of exposure showed they ranged from 5.7% to 21.6%, depending on the outcome and the chosen exposure variable. This suggests that, if indeed there is a causal relation, it is not trivial and that perhaps we should further study the problem and do more to integrate this into prevention. The intense desert dust episodes occurring during the third trimester seemed to be of particular interest.

Our study has a number of limitations. Contrary to the prospective cohort in Guadeloupe, ours is a retrospective ecological cohort. Given the ecological nature of the exposure, it is conceivable that exposure misclassification occurred if women moved to other areas during pregnancy or, more importantly, given the frequent poverty in French Guiana, if different living conditions led to differences in exposure (working outside, living in slums, etc.). It is also arguable that PM10 measurements from a single station in Cayenne may not give an accurate reflection of the actual exposure of individual women. However, the fact that desert dust episodes are prolonged and are visible on satellite photographs and to the naked eye shows that they occur at a scale well above that of the city of Cayenne (20) and that when an episode occurs, it generally affects all of the city, not just some focal areas. The pregnancy registry was filled out at delivery, and the PM10 measurements for each day of the pregnancy were inferred by the ATMO-Guyane daily measurements between the beginning of pregnancy and delivery. However, the outcome is clear, and the use of PM10 measurements constitutes a meaningful dataset. Furthermore, we adjusted for a number of potential confounders associated with preterm delivery. Nonetheless, other seasonal confounders aligned with desert dust episodes’ temporality cannot be ruled out. Selection bias is a theoretical risk, but the registry is exhaustive, and the analysis only retained all women living and delivering in Cayenne to avoid such a risk. Finally, the computation of population-attributable fractions with exposures for which causal relations with the outcome have not been proven could be criticized. However, here, we aimed to provide a sense of the magnitude of “impact” to determine whether the association is potentially meaningful from a public health perspective.

Overall, the highlights of the present study are that the highest mean concentrations and intense desert dust episodes were indeed associated with both spontaneous and induced preterm delivery, as observed in Guadeloupe. The most striking associations with increased preterm delivery were observed for the proportion of intense desert dust episodes, notably during the third trimester of pregnancy. However, in our study, the relation between PM10 concentrations appeared non-linear. Given the observed discrepancies between studies, further research in other sites affected by desert dust should also test the linearity of the relation between desert dust and preterm delivery. Whether this has any practical value for the follow-up of pregnant women is not clear. Although it is an untested idea, informing women to monitor ATMO-Guyane’s alerts (21), staying inside during intense desert dust episodes, or wearing face masks to filter particles (29) could be a potential concrete recommendation. The potential efficacy of such an intervention should, however, be prospectively tested.

## Data availability statement

The data analyzed in this study is subject to the following licenses/restrictions: the datasets may be shared upon reasonable request after clearance with the Commission Nationale Informatique et Libertés, which regulates the circulation of data in France. Requests to access these datasets should be directed to cicec@ch-cayenne.fr.

## Ethics statement

The studies involving humans were approved by Commission Nationale Informatique et Libertés. The studies were conducted in accordance with the local legislation and institutional requirements. Written informed consent for participation was not required from the participants or the participants’ legal guardians/next of kin in accordance with the national legislation and institutional requirements.

## Author contributions

MN and ML: conception. KP, KB, and FQ: data management. MN: data analysis and first draft. ML, CB, FQ, SB, AL, DD, LO, and NH: review and editing. All authors contributed to the article and approved the submitted version.
