# Supplementary figures and images for: Desert dust episodes during pregnancy are associated with increased preterm delivery in French Guiana
Source: Front Public Health. 2024 Feb 28;12:1252040. doi: 10.3389/fpubh.2024.1252040 (PMC10933026; doi:10.3389/fpubh.2024.1252040)

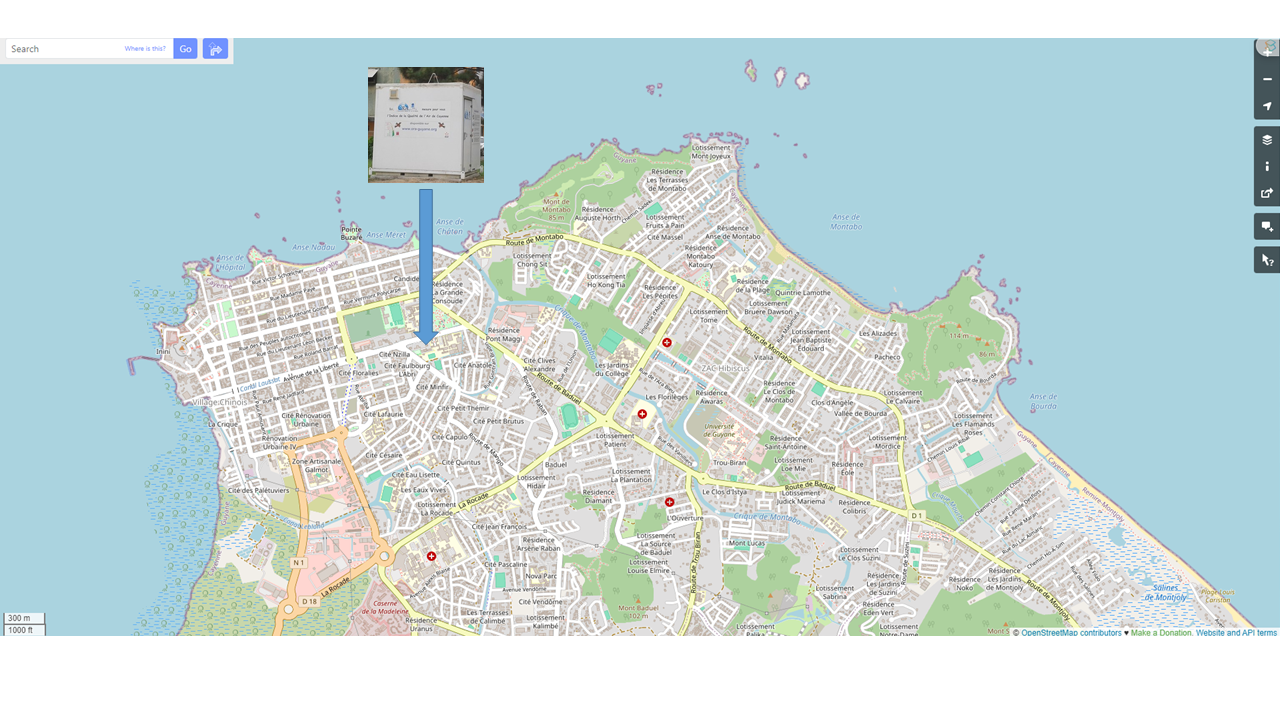

Supplement: Supplementary file 1 [file Image_1.TIF]
